# Thermal data from Málaga's historical centre: Surface and air temperature measurements captured via mobile station and thermal imaging

**DOI:** 10.1016/j.dib.2025.111274

**Published:** 2025-01-08

**Authors:** Giulia Forestieri, Francisco Tomatis, Daniel Jato-Espino, Monica Pena Acosta

**Affiliations:** a GREENIUS Research Group, Universidad Internacional de Valencia – VIU, Calle Pintor Sorolla 21, 46002 Valencia, Spain; b TADRUS Research Group, Department of Agricultural and Forestry Engineering, University of Valladolid, UVa Campus of Palencia, 34004 Palencia, Spain; c Department of Construction Management and Engineering, Faculty of Engineering Technology, University of Twente, PO BOX 217, 7500 AE Enschede, the Netherlands

**Keywords:** Thermal imaging, Surface temperature, Air temperature, Historical architecture, Urban microclimate

## Abstract

This dataset contains air and surface temperature measurements taken twice daily at 11:00 and 23:00 GMT+2 from 24th June to 5th July 2024 (a total of 10 days) in the historical centre of Malaga, Spain. It includes detailed thermal readings from various street materials and the facades of historical buildings, offering insights into the thermal properties and responses of these elements at different times of day. This dataset provides valuable information on localized temperature variations within the historical centre, influenced by different materials and architectural styles. It can be used to model microclimate variations, evaluate the thermal behavior of both historical and contemporary materials, and inform urban planning and heritage conservation efforts*.*

Specifications Table

**Table:** 

Subject	Environmental Science: Climatology.
Specific subject area	Urban microclimate analysis, thermal properties of materials, historical architecture temperature response.
Type of data	The dataset consists of quantitative environmental data and thermal images. The data types include:Tabular Data: • Air temperature, surface temperature, relative humidity, wet-bulb temperature, and dew point measurements, recorded at different times of day (day and night) across various streets. • Coordinates (latitude and longitude) for the start and end points of each street segment where measurements were taken. Image Data: • Thermal images of building facades and street surfaces were captured using a handheld infrared camera.
How the data were acquired	The data were collected using two parallel methods. First, a bicycle-based mobile station equipped with a thermologger was used to capture high-resolution surface and air temperature readings at 11:00 and 23:00 GMT+2 along 11 streets in Málaga's historical centre. Concurrently, a Fluke TI400 thermal camera captured thermal images of building facades, recording surface temperatures at various heights.
Data format	Dataset 1: CSV (Comma-Separated Values). Dataset 2: ZIP file.
Description of data collection	Data collection occurred between 24th June and 5th July 2024 in Málaga's historical centre. Air and surface temperatures were measured twice daily at 11:00 and 23:00 GMT+2 using a mobile station equipped with a thermologger. Additionally, thermal images of 11 building facades were taken using a handheld thermal camera, focusing on different street materials and architectural elements.
Data source location	City/Town/Region: Málaga, Andalusia Country: Spain Latitude and Longitude: Approx. 36.7213° N, 4.4213° W
Data accessibility	Repository name: Thermal Data from Málaga: Surface and Air Temperature via Mobile Station & Imaging Direct URL: 10.4121/20167a4c-b51c-422c-a30d-7580acc3125b.v1 DOI: 10.4121/20167a4c-b51c-422c-a30d-7580acc3125b.v1

## 1 Value of the Data

The dataset provides detailed air and surface temperature measurements within the historical centre of Malaga, offering critical insights into how urban materials and architecture influence localized temperature variations. By capturing the thermal behavior of historical buildings and streets, it contributes to understanding the thermal properties of different materials, which is valuable for both heritage conservation and urban planning.

• With twice-daily measurements, the dataset captures temperature variations across different times of day, allowing for the analysis of diurnal patterns in urban microclimates.
• Although not directly aimed at studying the urban heat island (UHI) effect, the dataset offers valuable inputs for future UHI analysis by focusing on densely built historical areas with complex thermal dynamics.
• By leveraging the dataset's information, researchers can analyze the thermal response to different strategies aimed at combating the rise of urban temperatures, particularly in densely built historical areas where material properties ansssssssd architectural styles significantly impact local microclimates.

## 2 Data Description

### 2.1 Dataset 1: mobile_station_temp_data.csv

Dataset 1 includes a CSV (Comma-Separated Values) file called “Mobile_Station_Tem_Data”. This format is used for storing temperature, humidity, and geographic data, including variables such as air temperature, surface temperature, relative humidity, wet-bulb temperature, dew point, and the start and end coordinates of each street segment. Table 1 contains temperature and humidity measurements collected via the mobile station in Málaga's historical centre between 24th June and 5th July 2024. The table includes the following columns: Street_ID (unique identifier for each street), Shift (Day/Night), Date, Start_time, End_time, Avg_Temp_Air (average air temperature in °C), Avg_Temp_Surface (average surface temperature in °C), Avg_RH (average relative humidity in %), Avg_WB (average wet-bulb temperature in °C), Avg_DP (average dew point in °C), Start_X and Start_Y (starting point coordinates), End_X and End_Y (ending point coordinates). Each record represents a temperature measurement for a specific street and time. Below is a detailed description of each field:

**Table 1:** Data content and format.

Field	Description	Unit	Example
Street_ID	Unique identifier for each street where measurements were taken. It corresponds to a specific street in the study area (e.g., historical streets in Málaga).	N/A	9 (for S. Augustín street)
Shift	Indicates whether the data was collected during the day or night.		Day: Data collected during the day (typically around 11:00 UTC)
Night: Data collected during the night (typically around 23:00 UTC).			
Date	The date on which the measurements were taken.		26 June 2024
Start_time	The time when data collection began for a particular street on the given date.	HH:MM (GMT+2)	23:15:00
End_time	The time when data collection ended for a particular street on the given date.		23:25:00
Avg_Temp_Air	Average air temperature measured along the street during the data collection period.	Degrees Celsius (°C)	27.21 °C (average air temperature during the measurement period)
Avg_Temp_Surface	Average surface temperature measured on street surfaces during the data collection period.		23.06 °C (average temperature of road, pavements during the measurement period)
Avg_RH	Average relative humidity (RH) recorded during the data collection period.	Percentage (%)	54.54 % (relative humidity in the air)
Avg_WB	Average wet-bulb temperature, which represents the lowest temperature that can be reached under current ambient conditions by the evaporation of water.	Degrees Celsius ( °C)	20.75 °C
Avg_DP	Average dew point temperature, which is the temperature at which the air becomes saturated with moisture and dew forms.		17.25 °C
Start_X	The X-coordinate (longitude) of the starting point of the street segment where measurements began.	Coordinate in the projection system being used (e.g., WGS84 for GPS coordinates)	−4.421312 (longitude of the starting point)
Start_Y	The Y-coordinate (latitude) of the starting point of the street segment where measurements began		36.721078 (latitude of the starting point)
End_X	The X-coordinate (longitude) of the ending point of the street segment where measurements ended.		−4.423122 (longitude of the ending point)
End_Y	The Y-coordinate (latitude) of the ending point of the street segment where measurements ended.		36.721828 (latitude of the ending point)

Table 2 provides a summary of the average air and surface temperatures, as well as the relative humidity recorded during day and night across various streets in Málaga's historical center. The table displays the following parameters for each street ID: Average air temperature during the day and night (°C); Average surface temperature during the day and night (°C). The data highlights the variations between daytime and nighttime temperatures.

**Table 2:** Summary of average air and surface temperatures and relative humidity for day and night across streets in Málaga's historical centre.

Street_ID	Avg_Temp_Air_Day (°C)	Avg_Temp_Air_Night (°C)	Avg_Temp_Surface_Day (°C)	Avg_Temp_Surface_Night (°C)
1	26.79	25.28	24.77	26.74
2	26.51	25.29	27.16	28.15
3	27.02	24.91	26.97	23.99
4	27.38	25.01	31.01	26.77
5	26.47	24.88	25.48	24.02
6	27.16	24.88	30.07	25.66
7	26.83	24.95	26.11	23.81
8	26.81	25.07	31.64	26.11
9	26.91	25.14	24.41	24.99
10	26.74	25.32	24.68	23.85
11	27.45	25.25	33.52	28.17

**Fig. 1:**
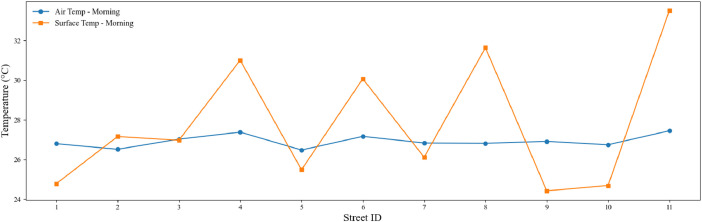
Morning air and surface temperature variation across the 11 streets that were surveyed. The graph presents the average air temperature (°C) and surface temperature (°C) measured during daytime hours.

**Fig. 2:**
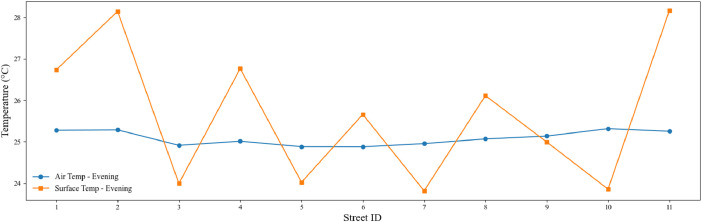
Evening air and surface temperature variation across the 11 streets that were surveyed. The graph presents the average air temperature (°C) and surface temperature (°C) measured during the evening data collection campaign.

Fig. 1, Fig. 2 depict the variation in air and surface temperatures across the surveyed streets during the daytime and the nighttime, respectively. The blue line represents the air temperature, while the orange line corresponds to the surface temperature.

### 2.2 Dataset 2: thermal_images.zip

Dataset 2 is a ZIP format named "thermal_images" which contains two folders and two files:

1. Folder: "Building reports": it includes subfolders, each containing .pdf reports of the buildings and pavements. Subfolders are named according to the street ID, measurement date, time of capture (day/night) and the name of the building. The reports provide detailed thermal measurements for each building and pavement, with a focus on specific areas as described in the methodology.
2. Folder: "FC Cloud": it contains thermographic images captured by the Fluke TI400 handheld thermal camera in IS2 format. Each file corresponds to the thermal images taken during the field campaign, capturing surface temperatures of building facades and pavements.
3. CSV file named "Building thermal measurements": it contains the average temperature data of the buildings at three different points of measurement on the facades: the lowest point near the street, the middle section, and the highest point. The data are organized by building ID and timestamp, allowing for easy comparison across different structures and times of day.
4. CSV file called "Pavements measurements": this .csv file provides temperature data for the various pavements measured during the fieldwork. Each entry is categorized by pavement type, street ID, and the time of measurement, offering insights into the thermal properties of different urban surface materials.

Table 3 contains average temperature measurements collected via the handheld thermal camera during the campaign period. The table includes the columns reported in both .csv files. In the first CSV file there are the following columns: Street_ID; Building ID; Building name; date; Shift (Day/Night); average building temperatures in °C for the low (Avg_Temp_low), medium (Avg_Temp_Medium), high (Avg_Temp_High), and top (Avg_Temp_Top) parts of the facade, as well as the average for the entire building (Avg_Temp_Building). In the second .csv file, "Building pavements.csv," there are additional columns that differ from the first file, including the type of the street material (street material) and its temperatures (Temp_street_material (°C)).

**Table 3:** Data content and format.

Field	Description	Unit	Example
Street_ID	The same as Dataset 1.	N/A	See Table 1.
Building_ID	Unique identifier for each building where measurements were taken. It corresponds to a specific building in the study area.		See Table 6.
Building_name	The name of each measured building.		See Table 6.
Street material	The name of the street material surrounding the measured buildings.		See Table 7.
Shift	The same as Dataset 1.		See Table 1.
Date	The same as Dataset 1.		See Table 1.
Avg_Temp_low; Avg_Temp_medium; Avg_Temp_high; Avg_Temp_top	Average air temperatures measured along the building facade during the data collection period at the low, medium, high, and top sections of each facade.	Degrees Celsius (°C)	30.80 °C (average low building temperature during the measurement period)
Avg_Temp_buidling	Average building temperature		28.40 °C (average building temperature during the measurement period)
Temp_street_material	Average street material		36.90 °C (average street material during the measurement period)

Table 4, Table 5 present the average temperature values measured on building facades at three vertical levels (low, medium, and high) and on street pavements. Table 4 summarizes the daily temperature differences, while Table 5 focuses on nightly temperature variations. The measured temperatures are represented as averages for each building façade and pavement material.

**Table 4:** Average daily temperature differences across facade levels and pavement materials.

Building_ID	Avg_Temp_low (°C)	Avg_Temp_medium (°C)	Avg_Temp_high	Avg_Temp_top (°C)	Avg_Temp_buidling (°C)	Temp_pavement_material (°C)
1	27.23	28.47	27.96	N/A	27.89	31.32
2	27.45	26.75	25.68	28.20	26.64	31.78
3	27.57	30.15	30.74	30.50	29.53	27.09
4	26.36	26.70	26.32	N/A	26.46	36.68
5	31.69	32.73	27.83	N/A	30.75	34.53
6	28.78	27.68	26.17	26.15	27.32	34.67
7	27.94	27.02	27.18	27.20	27.33	31.48
8	27.71	28.26	27.15	27.30	27.62	37.49
9	27.00	27.40	27.33	29.30	27.27	31.41
10	29.45	29.29	28.62	27.98	28.80	28.77
11	32.61	29.43	32.40	N/A	30.84	37.48

Cells marked as N/A indicate monitoring gaps due to obstructions in data collection at specific heights or time periods.

**Table 5:** Average nightly temperature differences across facade levels and pavement Materials.

Building_ID	Avg_Temp_low (°C)	Avg_Temp_medium (°C)	Avg_Temp_high	Avg_Temp_top (°C)	Avg_Temp_buidling ( °C)	Temp_pavement_material (°C)
**1**	25.49	24.26	24.50	24.00	24.84	25.19
**2**	25.79	24.00	24.74	23.05	24.96	30.61
**3**	27.40	26.04	26.10		26.51	29.20
**4**	26.31	26.10	26.26	N/A	26.23	29.80
**5**	25.96	25.46	24.91	22.80	25.39	27.61
**6**	26.14	25.33	25.25	24.05	25.46	26.63
**7**	26.73	29.51	27.61	25.97	27.81	29.83
**8**	27.15	25.46	25.86	25.22	26.04	30.86
**9**	26.43	25.98	26.13	25.54	26.08	29.46
**10**	26.64	27.34	26.31	25.87	26.64	28.42
**11**	26.07	25.13	N/A	N/A	25.60	30.96

Cells marked as N/A indicate monitoring gaps due to obstructions in data collection at specific heights or time periods.

### 2.3 Table 6: street and building data nomenclature

This table provides the mapping of Street_IDs and Building_IDs to specific locations within Málaga's historical centre. It lists the corresponding street names, building names, and descriptions of each building (e.g., historical significance, architectural features).

### 2.4 Fig. 5: route of data collection

This figure illustrates the route followed during data collection in Málaga's historical centre. It shows the 11 streets, and 11 historical buildings surveyed, with streets marked in black lines and building locations indicated by red circles. The route is based on GPS coordinates and represents the spatial context of the dataset (Fig. 4).

**Fig. 3:**
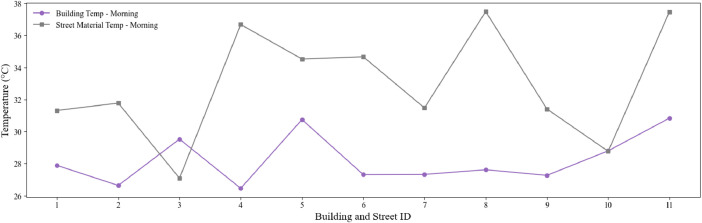
Morning façade and pavement material temperature profiles across buildings and pavements. The plot highlights the average building façade temperature (°C) and corresponding pavement material temperature (°C) recorded during the data collection campaign.

**Fig. 4:**
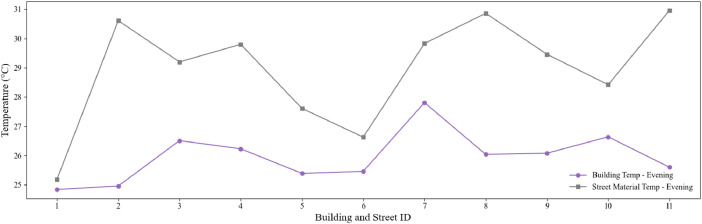
Evening façade and pavement material temperature profiles across buildings and pavements. The plot highlights the average building façade temperature (°C) and corresponding pavement material temperature (°C) recorded during the data collection campaign.

### 2.5 Table 7: street construction materials

This table provides details on the materials used in street construction within the historical centre. It maps the Street_ID to the types of materials present, such as basalt, limestone, granite, cobblestones and others.

## 3 Background

**Fig. 5:**
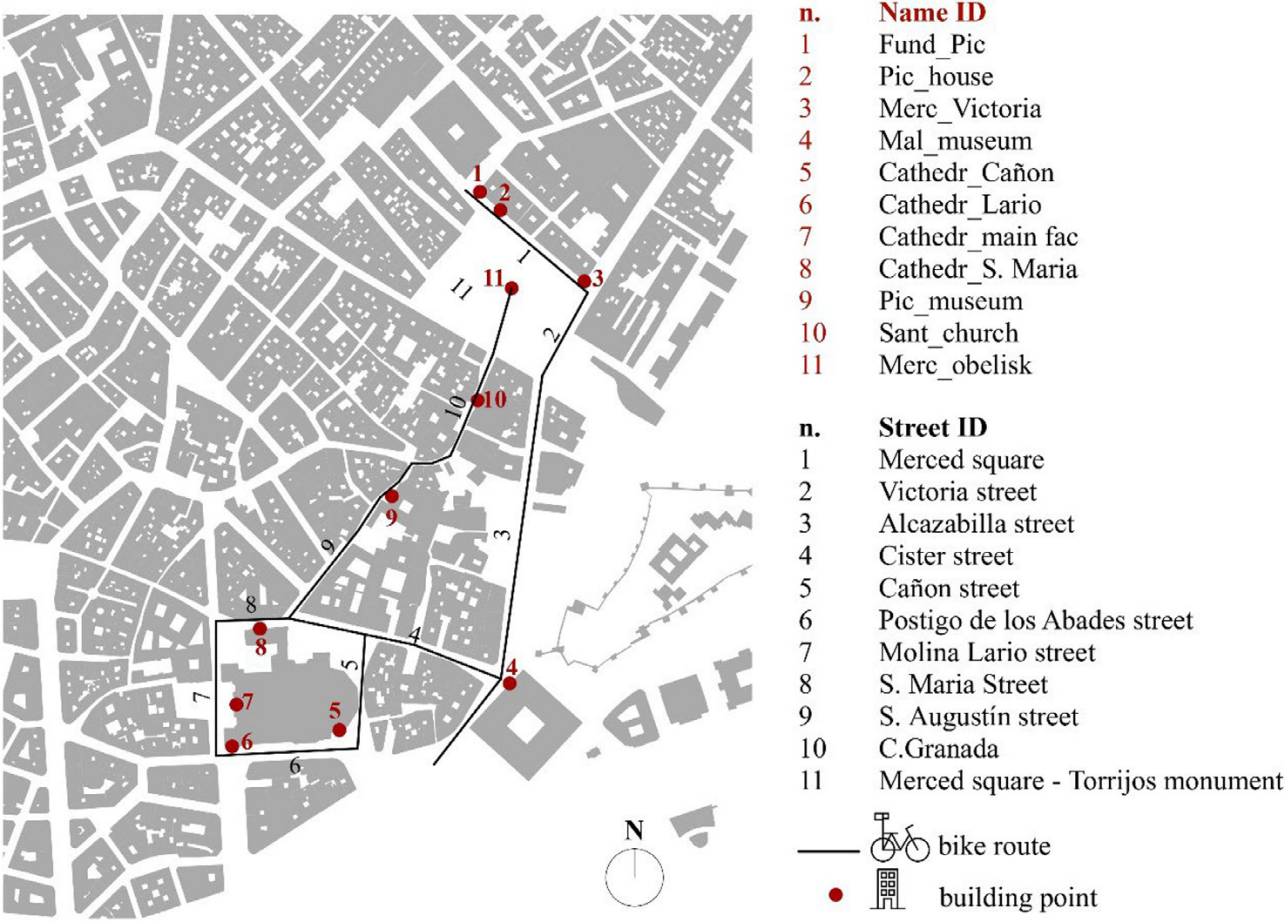
Route of the 11 streets and the 11 buildings measured in the historical center of Malaga. The buildings are marked with red circles, and the streets surveyed are indicated with black lines.

The motivation for this dataset arises from Málaga's efforts to integrate climate criteria into urban planning, as outlined in the *ALICIA* initiative of the city's Climate Plan [1]. Located in southern Spain, Málaga experiences a Mediterranean climate characterized by high summer solar irradiation and frequent periods of overheating [2]. Its historical centre, with dense construction and limited exposure to cooling marine breezes, creates a unique microclimate marked by elevated temperatures and pronounced urban heat island (UHI) effects. Málaga's historical centre is currently one of the main focal points for tourist activity, exerting huge pressure on architectural heritage. By providing precise data on the thermal properties of various streets along the Picassian Route and historical building facades (Table 6; Fig. 5), this dataset contributes to the scientific understanding of how urban morphology affects microclimates. It directly supports the broader goals of the Málaga Urban Agenda and the Covenant of Mayors for Climate and Energy by serving as a foundation for research into localized climate adaptation strategies, informing sustainable urban design, and guiding heritage conservation efforts in the face of rising global temperatures.

**Table 6:** Legend of street and building data nomenclature.

Street ID	Street name	Building number ID	Building name ID	Building description
1	Merced square	1	Fund_Pic	Picasso foundation
2	Victoria street	2	Pic_house	Picasso house
3	Alcazabilla street	3	Merc_Victoria	Intersection Merced Square - Victoria street
4	Cister street	4	Mal_museum	Malaga Museum
5	Cañon street	5	Cathedr_Cañon	Cathedral - Cañon Street
6	Postigo de los Abades street	6	Cathedr_Lario	Cathedral - Molina Lario Street
7	Molina Lario street	7	Cathedr_main fac	Cathedral - Bishop square
8	S. Maria Street	8	Cathedr_S. Maria	Cathedral - S. Maria Street
9	S. Augustín street	9	Pic_museum	Picasso Museum
10	Granada street	10	Sant_church	Santiago's church
11	Merced square - Torrijos monument	11	Merc_obelisk	Merced Obelisk "Torrijos" monument

### 3.1 Experimental Design, Materials and Methods

**Fig. 6:**
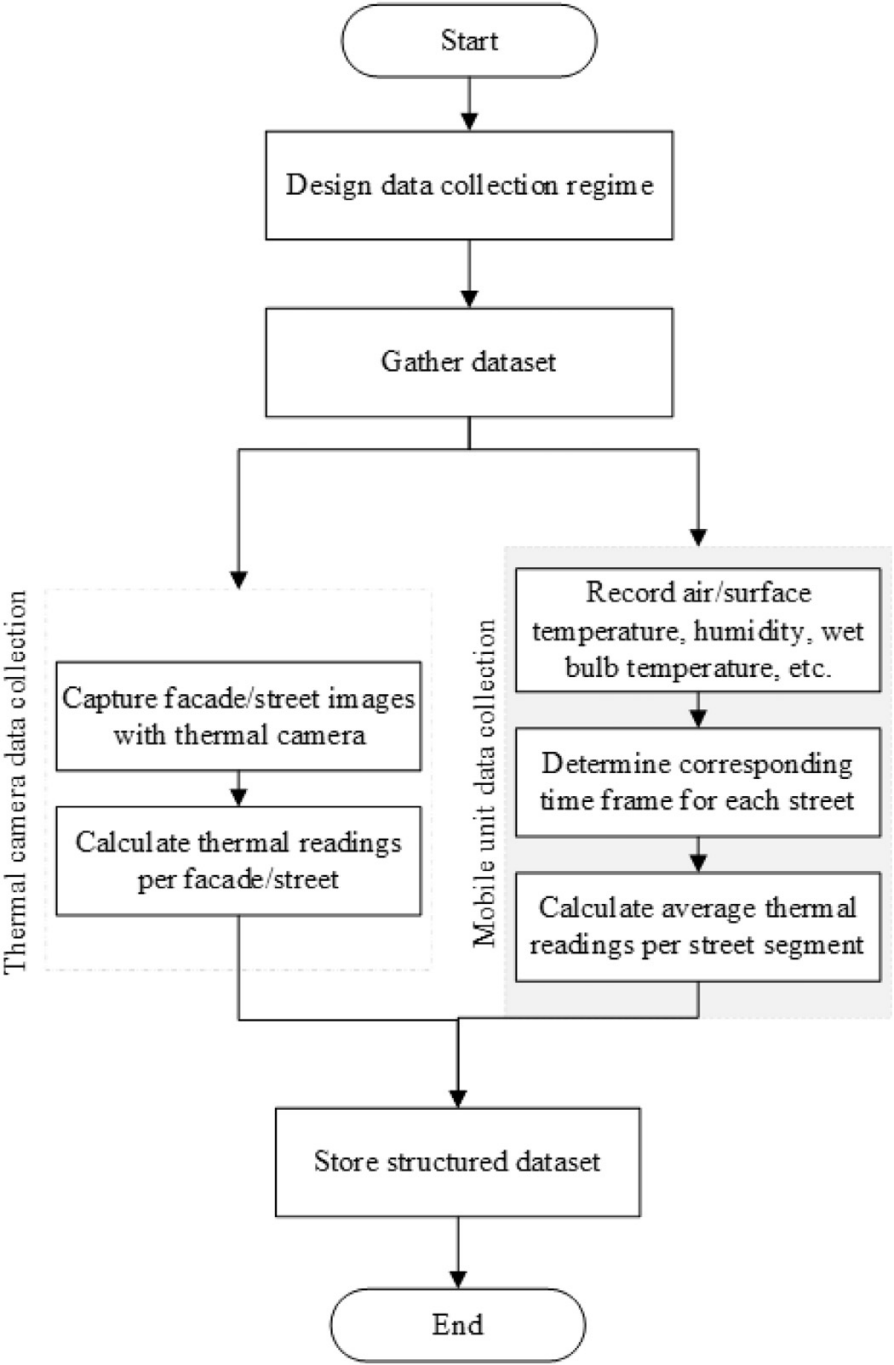
Process flow detailing how the data collection was organized and structured into a final dataset.

Data collection was carried out in two parallel processes. In the first process, a mobile unit [3] was used to traverse 11 streets in the historical centre of Malaga, capturing high-resolution air and surface temperature data, along with humidity and other thermal metrics at 11:00 and 23:00 UTC daily. The recorded variables included air temperature, surface temperature, relative humidity, wet bulb temperature, and dew point. Concurrently, the second process involved using a handheld Fluke TI400 thermal camera to capture thermal images of the facades of 11 historical buildings and the streets directly in front of them. The thermal data included maximum, minimum, and average temperatures of both the facades and street materials, as well as emissivity values. Together, these parallel processes generated a detailed dataset on both environmental and built-surface thermal characteristics across the study area. Fig. 6 provides an overview of the process flow, outlining the organization and structuring of data collection leading to the creation of the final dataset. The following sections provide a detailed description of the study area, and the instruments used for data collection.

### 3.2 Study area description

Málaga, Spain, is a historical city known for its vibrant old town, with narrow streets and ancient architecture dating from the 15th to the 19th century, as well as cultural landmarks such as Santiago's Church (1493–1545), the Cathedral (1527–1782), and the Torrijos Monument (1842). The historical centre is characterized by Mediterranean-style buildings constructed with stone block masonry and surface finishes in mortar, which absorb and retain heat. The materials used for street construction in the historical centre are predominantly limestone (in various colors), basalt, granite, cobblestones, pebbles, and asphalt on the surrounding vehicular roads (Table 7). The city experiences high temperatures that frequently exceed 30 °C, with peaks above 40 °C during heatwaves. In the summer of 2023, Málaga recorded a temperature of 43.3 °C, marking one of the highest on record [2]. The combination of heat retention by urban materials and limited airflow in the narrow streets amplifies the heat. These conditions make Málaga one of the European cities most impacted by rising temperatures.

**Table 7:** Legend of street construction materials.

Street material	Street ID	Building ID	Building name
Basalt	1	1	Fund_Pic
Cream Limestone	1	2	Pic_house
	3	4	Mal_museum
	5	5	Cathedr_Cañon
	6	6	Cathedr_Lario
	10	10	Sant_church
	11	11	Merc_obelisk
Asphalt	2	3	Merc_Victoria
Granite Cobblestones	2	3	Merc_Victoria
Cobblestones	3	4	Mal_museum
	7	7	Cathedr_main fac
Limestone	3	4	Mal_museum
	6	6	Cathedr_Lario
Rectangular Cobblestones	6	6	Cathedr_Lario
Granite	7	7	Cathedr_main fac
White Limestone	8	8	Cathedr_S. Maria
Gray Limestone	8	8	Cathedr_S. Maria
	10	10	Sant_church
Black Limestone	8	8	Cathedr_S. Maria
Multicolor Limestones	9	9	Pic_museum
Pebbles	9	9	Pic_museum

### 3.3 Instruments and data collection methods

As summarized in Fig. 6, two parallel methods were implemented to capture thermal data in the historical centre of Málaga. A bicycle-based mobile station equipped with a thermologger and a handheld thermal camera were employed to collect comprehensive air and surface-specific data.

### 3.4 Bicycle mobile station

**Fig. 7:**
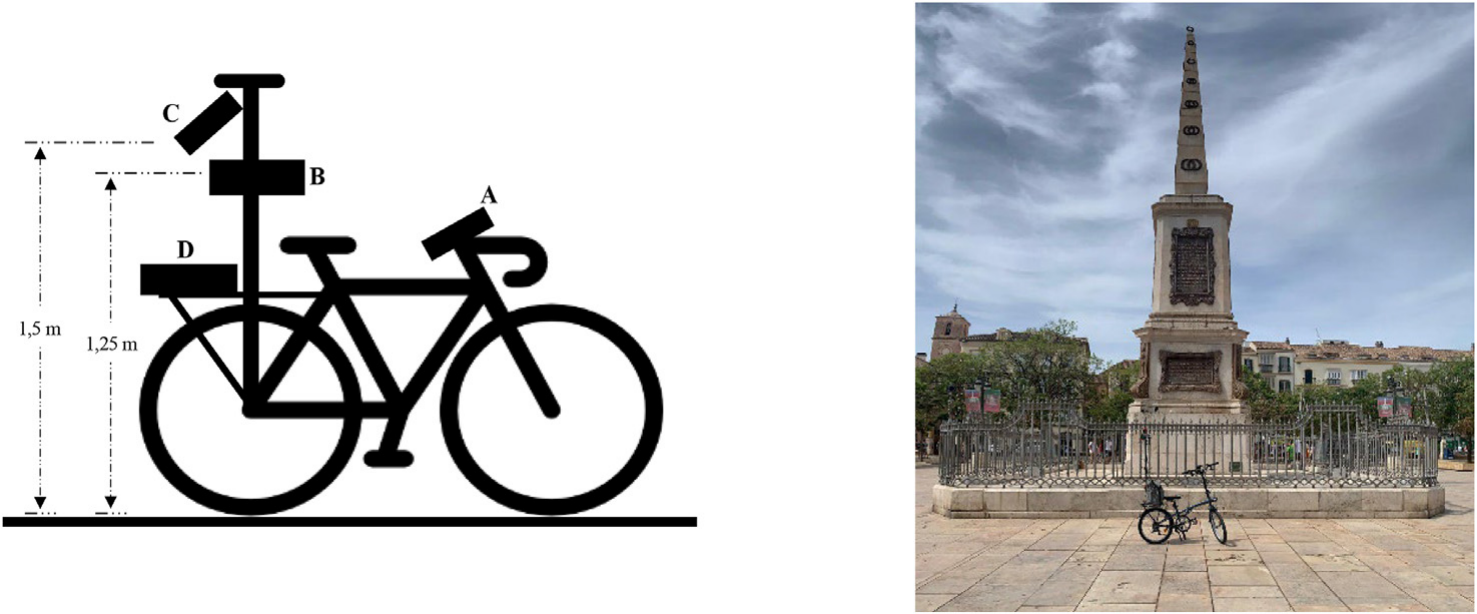
Left: A schematic of the bicycle-based mobile urban data-gathering station, illustrating the placement of key sensors relative to the ground. Adapted from [1]. Right: A photograph of the mobile station in use, positioned in the historical centre of Málaga, highlighting its field deployment for temperature data collection.

A bicycle-based mobile station (Fig. 7) was used to measure air temperature, surface temperature, and relative humidity along a predefined route covering 11 streets. The thermologger captured high-resolution data in real time, monitored via a display, and managed through a Windows-based data acquisition system. Despite the street canyon's complexity, a GPS rover was not utilized; instead, time markers were employed to correlate the data to specific locations along the route. This approach allowed precise alignment of the recorded temperatures with the exact street segments (Table 8).

**Table 8:** Sensor overview.

Sensor	Reference in Fig. 3	Function	Model	Measured parameter
Display	A	Monitor the campaign in real time	N/A	N/A
IR Camera	B	Façade temperature monitoring	FLIR A45 FOV 69	Urban morphology temperatures
Thermologger	C	Surface temperature monitoring and weather station	Extech HD500	Surface Air Temperature, Relative Humidity, Wet Bulb
Processing Centre	D	Data Processing	Microsoft surface pro-2	N/A

This table provides an overview of the sensors and devices used in the bicycle mobile station, along with their respective functions and measured parameters.

### 3.5 Handheld thermal camera

**Fig. 8:**
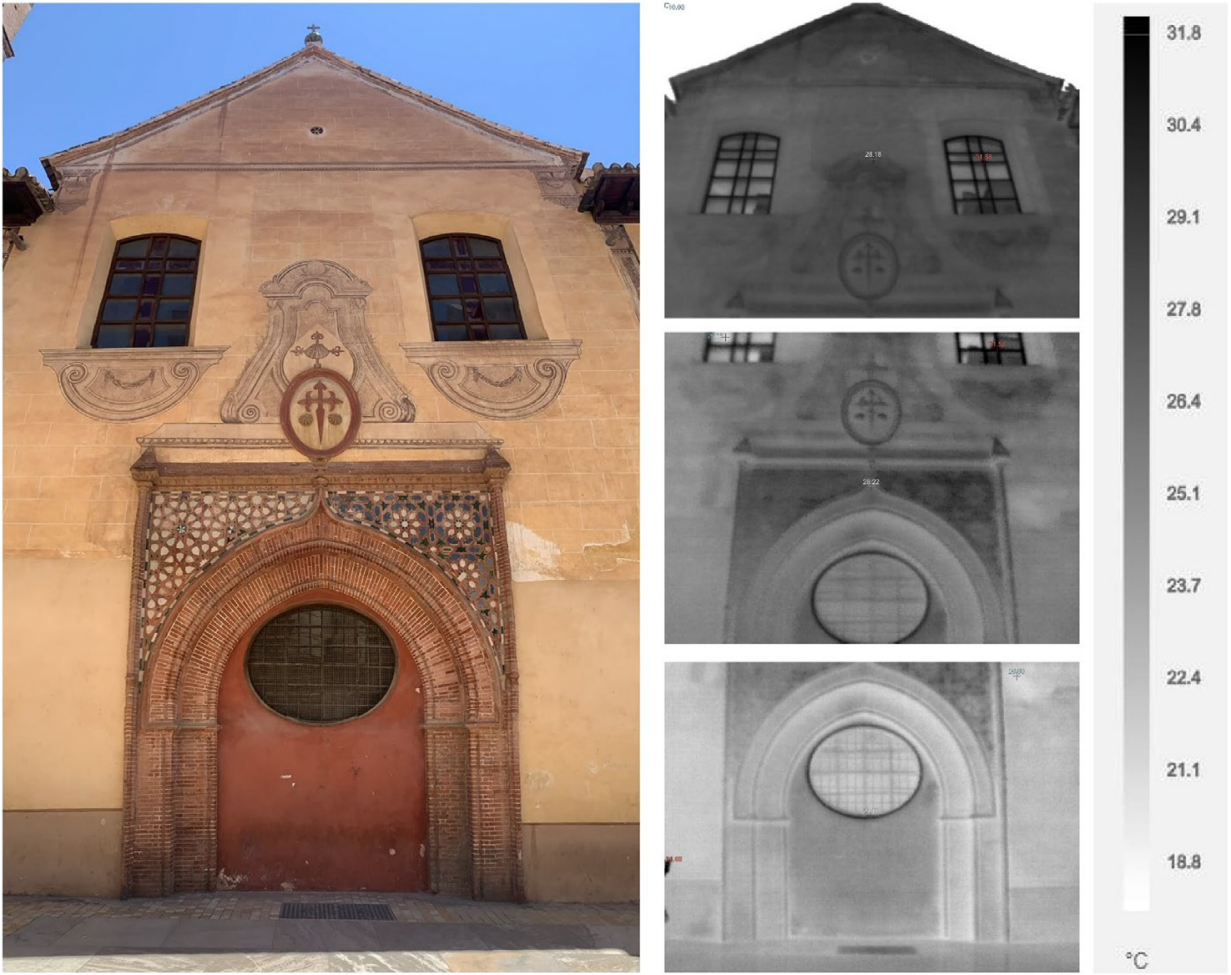
The image on the left shows a photograph of Santiago's church (built during 1493–1545). The three thermal images on the right, presented in grayscale, show the facade's surface temperature distribution at different vertical points: from the lowest point near street level (bottom), the midpoint of the facade (middle), to the highest point (top).

**Fig. 9:**
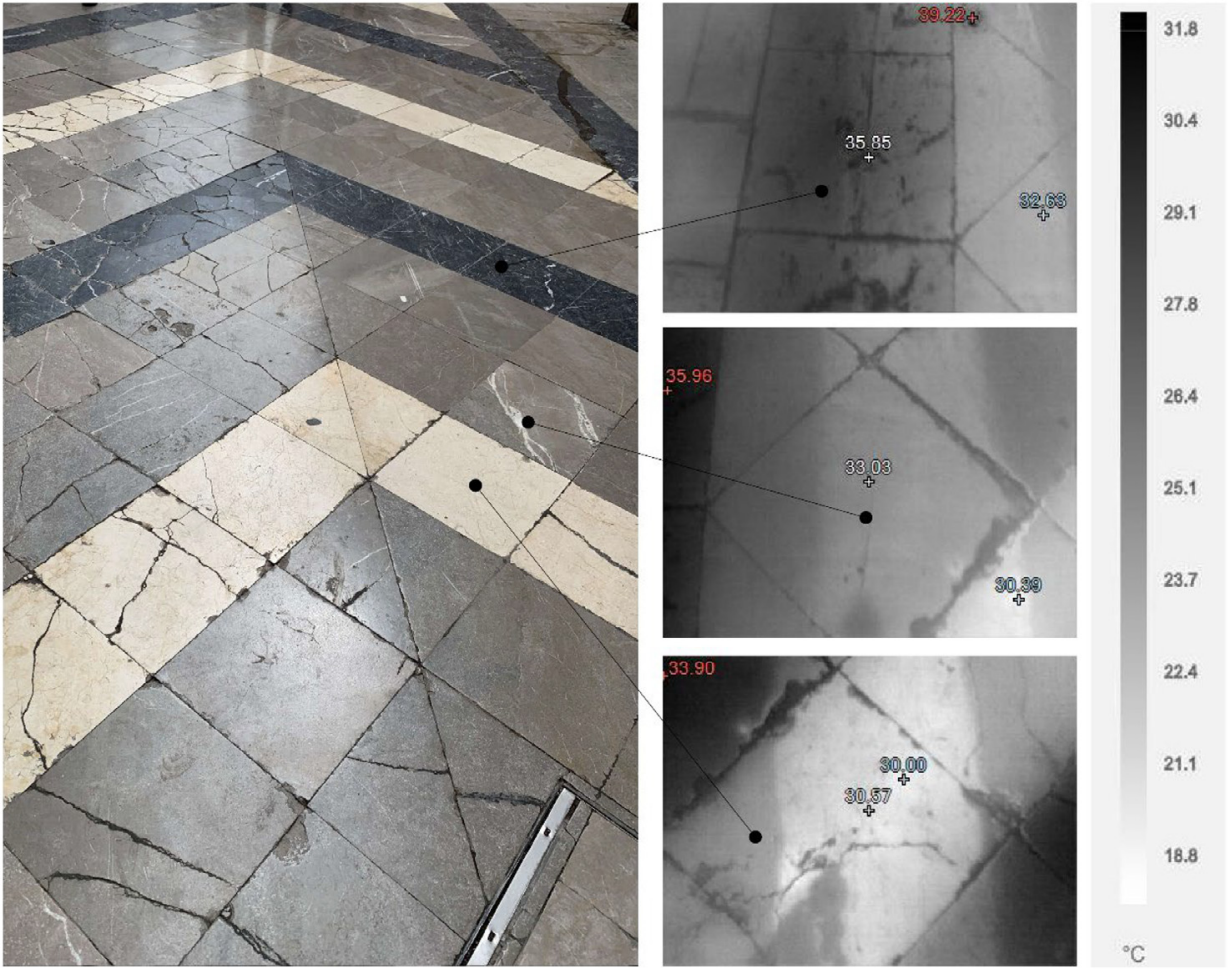
The image on the left depicts S. Maria Street, where the pavement is made up of limestone in three colors: white, gray, and black. The three thermal images on the right, presented in grayscale, show the corresponding temperatures for each color: the white limestone (bottom) records 30 °C, the gray (middle) 33 °C, and the black (top) 35 °C.

In parallel, a Fluke TI400 handheld thermal camera was used to capture thermal images of building facades. The measurement parameters were an emissivity of 0.92, a background temperature of 20 °C, a calibration range from −20 °C to 80 °C, and an IR sensor size of 320 × 240. Thermal camera data collection was conducted simultaneously with mobile measurements using bicycle, during the same period from June 24th to July 5th, 2024. The facades of historical buildings were selected as key points of interest, and thermal readings were taken at three vertical levels: street level, the midpoint, and the top of each facade. The different types of pavements in front of the buildings were also measured with thermal camera to better understand heat absorption and emission, which might have implications for thermal efficiency in historical areas. This technique provided vertical thermal profiles of the structures (see Fig. 8), offering insights into how heat is distributed across different heights of the buildings and construction street materials (see Fig. 9).

## Limitations

Although this dataset offers valuable insights into the thermal behavior of Málaga's historical center, there are some limitations to consider. The data collection was conducted over a short period, from June 24th to July 5th, 2024, focusing exclusively on summer conditions. While this captures critical thermal behaviors during the hottest time of the year, future studies could benefit from extending the data collection across different seasons to understand year-round urban climate dynamics. In terms of methodology, the use of a predefined route and fixed measurement times (11:00 and 23:00 GMT+2) means that temperature variations at other times of day, particularly during peak heat hours in the afternoon, were not captured. Including additional time points in future data collection could provide a more comprehensive thermal profile of the area. The thermal camera measurements also faced minor constraints due to the narrow streets typical of Málaga's historical center. The varying distance between the camera and building facades may have slightly affected the precision of the surface temperature readings. However, this limitation is inherent to the complex urban geometry and is unlikely to have significantly impacted the overall dataset quality. Despite these considerations, the dataset remains a robust resource for urban thermal analysis and serves as a strong foundation for further research into localized climate behaviors in historical urban environments.

## Ethics Statement

The authors have read and follow the ethical requirements for publication in Data in Brief and confirming that the current work does not involve human subjects, animal experiments, or any data collected from social media platforms.

## CRediT Author Statement

**Giulia Forestieri:** Funding acquisition, Conceptualization, Investigation, Writing- Original draft preparation, Writing- Reviewing and Editing. **Francisco Tomatis:** Investigation, Resources. **Daniel Jato Espino**: Conceptualization, Investigation, Validation. **Mónica Peña Acosta**: Conceptualization, Methodology, Data curation, Investigation, Writing- Original draft preparation, Writing- Reviewing and Editing, Resources.

## Data Availability

4TU.ResearchDataThermal Data from Málaga: Surface and Air Temperature via Mobile Station & Imaging (Original data).
